# Statistical models of morphology predict eye-tracking measures during visual word recognition

**DOI:** 10.3758/s13421-019-00931-7

**Published:** 2019-05-17

**Authors:** Minna Lehtonen, Matti Varjokallio, Henna Kivikari, Annika Hultén, Sami Virpioja, Tero Hakala, Mikko Kurimo, Krista Lagus, Riitta Salmelin

**Affiliations:** 1 Center for Multilingualism in Society Across the Lifespan, Department of Linguistics and Scandinavian Studies, University of Oslo, Oslo, Norway; 2 Department of Psychology, Åbo Akademi University, Turku, Finland; 3 Department of Psychology and Logopedics, Faculty of Medicine, University of Helsinki, Helsinki, Finland; 4 Department of Signal Processing and Acoustics, School of Electrical Engineering, Aalto University, Espoo, Finland; 5 Department of Neuroscience and Biomedical Engineering, School of Science, Aalto University, Espoo, Finland; 6 Centre for Social Data Science, Faculty of Social Sciences, University of Helsinki, Helsinki, Finland

**Keywords:** Eye movements, Lexical processing, Word recognition, Psycholinguistics, Mental models

## Abstract

We studied how statistical models of morphology that are built on different kinds of representational units, i.e., models emphasizing either holistic units or decomposition, perform in predicting human word recognition. More specifically, we studied the predictive power of such models at early vs. late stages of word recognition by using eye-tracking during two tasks. The tasks included a standard lexical decision task and a word recognition task that assumedly places less emphasis on postlexical reanalysis and decision processes. The lexical decision results showed good performance of Morfessor models based on the Minimum Description Length optimization principle. Models which segment words at some morpheme boundaries and keep other boundaries unsegmented performed well both at early and late stages of word recognition, supporting dual- or multiple-route cognitive models of morphological processing. Statistical models based on full forms fared better in late than early measures. The results of the second, multi-word recognition task showed that early and late stages of processing often involve accessing morphological constituents, with the exception of short complex words. Late stages of word recognition additionally involve predicting upcoming morphemes on the basis of previous ones in multimorphemic words. The statistical models based fully on whole words did not fare well in this task. Thus, we assume that the good performance of such models in global measures such as gaze durations or reaction times in lexical decision largely stems from postlexical reanalysis or decision processes. This finding highlights the importance of considering task demands in the study of morphological processing.

## Introduction

Processing of morphologically complex words (e.g., screen+ing+s) is an active topic in visual word recognition research. Studies on morphological processing have focused on determining whether complex words are recognized by decomposing them into their morphological constituents or whether they are stored as holistic units in our mental lexicon. A variety of cognitive models have been proposed which span from full decomposition models (e.g., Taft, 1979, 2004), assuming that all words are represented as morphemes, to full form models that claim that all known words are initially accessed via their whole-word representations (e.g., Butterworth, 1983). In addition, there are dual/multiple-route models (e.g., Schreuder & Baayen, 1995; Kuperman, Schreuder, Bertram, & Baayen, 2009) which assume that the mental processing system may include both types of representations and utilize different kinds of information in order to process words effectively. Processing of morphologically complex words has been studied by utilizing various tools such as reaction time (RT) measurements in visual word recognition tasks, tracking of eye-movements during reading, and techniques measuring brain activity elicited by visual or auditory presentation of words.

Furthermore, the temporal order in which these kinds of representations become active during visual word recognition has been subject to debate (see, e.g., Rastle & Davis, 2008; New, Brysbaert, Segui, Ferrand, & Rastle, 2004, Giraudo & Grainger, 2003a, 2003b). For example, a widely held view states that morphologically complex words are segmented to their constituents at early stages of word recognition (see, e.g., Rastle & Davis, 2008, for a review). At a later stage in which the semantic and syntactic features are accessed, these decomposed parts are then assumedly recombined to form a meaningful whole (Schreuder & Baayen, 1995; Taft, 2004). This stage can thus be sensitive to full-form measures such as surface frequency even if decomposition has taken place, i.e., they would reflect recombination of the morphemes already segmented at earlier levels of processing (Taft, 2004; Fruchter & Marantz, 2015). Previous eye-tracking research on recognition of morphologically complex words has revealed effects of both the whole words and the morphological constituents (e.g., Andrews, Miller, & Rayner, 2004; Hyönä, Bertram, & Pollatsek, 2004). In compound words, whole-word frequency effects have been observed earlier in time than effects of the constituents (Kuperman et al., 2009), challenging the obligatory early decomposition accounts observed in, e.g., lexical decision (e.g., Taft, 2004; Rastle & Davis, 2008). The present study aims to better understand the processes and representations accessed at different stages of word recognition. To do this, we study how different computational models that are based on different kinds of representational units correspond to measures of participants’ eye-movement behavior during visual word recognition.

One central theme in morphological processing studies has been the issue of optimization, i.e., determining the most optimal units of representation in the mental lexicon, in terms of minimizing both storage capacity and processing speed (Schreuder & Baayen, 1995). Finnish, for example, is a morphologically rich language where each noun has about 150 paradigmatic forms, and various clitic particles can additionally be attached to these forms. Storing all these word forms as whole units is thus unlikely to be economical for the storage capacity of the mental lexicon, suggesting that decomposing them into morphological constituents is a useful strategy for the cognitive system. However, inflected Finnish words robustly elicit longer RTs, larger error rates, and longer eye-fixations than matched monomorphemic words (Niemi, Laine, & Tuominen, 1994; Hyönä, Laine, & Niemi, 1995; Bertram, Laine, & Karvinen, 1999; Lehtonen & Laine, 2003), suggesting that decomposition may also entail a cost. It is, however, unclear what an optimal balance between these two costs is and whether it differs in early vs. later stages of word recognition.

Computational models can provide useful means for addressing issues related to optimization. In contrast to psycholinguistic models that are typically descriptive, the output of computational models is quantitative. It can therefore be directly compared to continuous performance measures such as RTs in a word recognition task or eye-tracking measures during reading. If a computational model is able to successfully predict variation in these cognitive measures, it is likely able to tell us something essential about the cognitive operations relevant in these tasks. Previous work on statistical modeling of morphological processing has utilized a variety of approaches, many of which have not assumed morphemes themselves to have an influential role in word processing. Such approaches include the distributed-connectionist models (Seidenberg, 2005; McClelland, 1988; Gonnerman, Seidenberg, & Andersen, 2007, see Rueckl, 2010 for a review) and the amorphous Naïve Discriminative Reader model (Baayen, Milin, Filipovic, Hendrix, & Marelli, 2011; Baayen, Shaoul, Willits, & Ramscar, 2016), which maps orthographic or phonetic input units directly to symbolic semantic units, without hidden units or a morphological level. Here, in contrast, we focus on models that allow morphological information to be utilized in storage of words and models that are based on the principle of optimization, a principle that is likely to bear relevance in the human cognitive system.

Morfessor (Creutz & Lagus, 2007) is a computational model that is able to learn segmentations of words in an unsupervised manner from unannotated data, and it applies a principle of optimization in building a concise lexicon of morphs. First, it stores word forms as wholes (assuming one word is one morph, e.g., *dog*, *dogs*). Then it utilizes these stored morphs in segmenting new incoming words (e.g., after storing *dog*, also − *s* gets stored separately when encountering the word *dogs*). Morfessor searches for a segmentation that is simultaneously compact and an accurate representation of the data. As an illustration, an extremely compact lexicon would include only letters but it would not provide a good representation of the data, whereas listing all words as whole units in the lexicon would be a very accurate but not compact representation of the data. Via the cost function based on the two-part coding scheme of the Minimum Description Length (MDL) principle (Rissanen, 1978), Morfessor attempts to find an optimal balance between the two. The first part in the cost function represents a cost for storage for the lexicon where larger units are more costly. The second part, in turn, represents a cost for computation where holistic units reduce the cost. If only the second part was included, all words would be stored as full forms, and this would be a problem, e.g., when encountering words with novel combinations of known morphemes.

In Morfessor, it is also possible to manipulate the emphasis the model places on these parts, or decomposed vs. full-form units. This can be done by manipulating a hyperparameter alpha, which enables one to vary the length of the units that Morfessor tends to produce. A small value of the hyperparameter provides a lexicon of short units (or morphs that the model stores), whereas a large value provides a lexicon of long units. As an example of extremes, Morfessor with an alpha value of 0.01 leads to a lexicon of units which largely resemble linguistically analyzed morphs, whereas an alpha value of 10 includes a lexicon of full forms (Virpioja, Lehtonen, Hultén, Kivikari, Salmelin, & Lagus, 2018). This feature allows us to investigate, within the same model type, whether a solution that decomposes words at practically all morpheme boundaries corresponds better to human word-recognition measures than one that keeps some or all boundaries unsegmented. Unsupervised models such as Morfessor utilize general learning principles in extracting regularities from the input and can in this way mimic the kind of human learning in which discovering regular structures and patterns from the linguistic environment is central. An interesting comparison point is provided by supervised models trained on pre-given linguistically structured input, for which parallels can be found in human learning with innate constraints.

Morfessor was initially studied in psycholinguistic context by Virpioja, Lehtonen, Hultén, Salmelin, and Lagus (2011b) who demonstrated that the self-information values predicted by Morfessor correlated highly with word recognition times for morphologically complex Finnish words in a visual lexical decision task. These correlations were higher for Morfessor than for typically used psycholinguistic variables, such as lemma frequency, length, or morphological family size. Following this first investigation, Virpioja et al., (2018) utilized Morfessor and other statistical models based on self-information in studying the optimal balance of storage and decomposition in the human mental lexicon. They used simple statistical models of morphology that are based on different representational units: words thoroughly decomposed based on their linguistic analysis, full word forms, and a solution which segments words at some morpheme boundaries and leaves others unsegmented. They compared these models’ predictions with lexical decision RTs and aimed to uncover whether human representations for morphologically complex words are based on decomposed morphemes, full forms, or something in-between. The best correspondence was found by using a combination of two models: an instance of Morfessor that segments words at some morpheme boundaries while not others (Morfessor with an alpha value of 0.8), and a whole-word model. While Morfessor does not incorporate information about different types of morphemes, the output segmentations differ to some extent for words carrying different type of morphemes. In the analysis of Virpioja et al., (2018), the best-performing Morfessor instance left most derivational morpheme boundaries unsegmented (in line with previous behavioral studies on derivational processing, e.g., Niemi et al., 1994; Bozic and Marslen-Wilson, 2010; Laudanna, Badecker, & Caramazza, 1992) whereas all clitic particles were kept separate from the rest of the word. Interestingly, it also left a large proportion of the inflectional suffixes unsegmented. The results were interpreted to support dual-route accounts of morphological processing.

As the Virpioja et al., (2011b, 2018) studies were based on lexical decision RTs, it is unclear whether the good performance of Morfessor and the whole-word model stem from particular, possibly different stages of the word recognition process. Word recognition times in a lexical decision task necessarily include several stages, including form-level (e.g., letter and bigram) processing and access to more abstract lexical representations (e.g., whole words or morphemes) but also decision-making processes and button-press-related motor preparation. Tracking of eye-movements during reading can be used to study increasingly automatic aspects of the process. It provides us with measures that allow a look on the processes at play also during word recognition, enabling an improved temporal resolution. First fixation duration (FFD) is an eye-tracking measure expected to reflect early stages of word recognition, while more global measures such as gaze duration (GD; sum of duration of all fixations on the word) are assumed to emphasize also later processing stages (see, e.g. Hyönä et al., 1995). In addition to these well-established measures, we also include a further measure of the later stages, namely gaze duration minus first fixation duration (GmF), to more closely focus on the processes taking place after the initial landing of the eyes on the word.

Using these measures, we aim to better understand whether the predictive power of unsupervised Morfessor in lexical decision (Virpioja et al., 2011b, 2018) stems primarily from early or late word recognition processes. We investigate how the MDL-based optimization principle of Morfessor and its different model variants (e.g., those that decompose words exhaustively vs. those that keep many words unsegmented) perform in predicting the different eye-tracking measures during word recognition. Our first aim is thus to study the question of optimal units of processing utilized at different processing levels, for a variety of morphologically complex (inflected and derived) words. We compare the relative performance of statistical models that are based on different kinds of representational units, e.g., those close to linguistically defined morphemes, full forms, or a solution which finds an optimal balance between the two: for some morpheme combinations this may be full forms and for some decomposed representations. To study the optimal balance between the two extremes, we vary the hyperparameter alpha in the first type of the Morfessor method, Morfessor Baseline. We compare these three Morfessor instances to a similar simple model which is, however, trained using linguistically pre-segmented input in a supervised manner and thus fully morpheme-based (Morph unigram model), and to a full-form model based on surface frequencies (Word unigram model). With this approach and the temporal dimension provided by eye-tracking, we aim to study the sensitivity of early vs. late word recognition processes to morpheme-based vs. more holistic units.

Our second aim is to investigate statistical models which predict upcoming morphological information on the basis of previously observed morphs. We investigate to what extent these kinds of predictive processes are used in online recognition of morphologically complex words. We hypothesize that information of the morpheme context is to some extent utilized in recognition of multimorphemic words, at least after initial landing of the eyes on the word and after accessing the first morphological constituent. An unsupervised model type that allows testing the effect of morpheme context is Morfessor Categories-MAP (CatMAP) (Creutz & Lagus, 2005a, 2007) that incorporates rudimentary structural information regarding word formation, i.e., that words may include prefixes, stems, and suffixes. The segmentations provided by the CatMAP method correspond in most cases more accurately to linguistic morph segmentations than the segmentation given by the Morfessor Baseline algorithm (Creutz & Lagus, 2007). However, there are still differences compared to the linguistically defined morphemes. Therefore, as a comparison, we investigate the performance of a supervised model (Morph bigram model) that also predicts upcoming morphs on the basis of previous ones in the same word but the model is during its training given linguistically pre-segmented input, i.e., it utilizes units that strictly correspond to linguistic morphemes.

Our focus is on computational models that provide self-information estimates. The measure of self-information or “surprisal” is the negative logarithm of the word’s probability estimated by a statistical language model and is a measure of how unexpected a word form is. This measure has previously been used, e.g., in the context of auditory word recognition (Balling & Baayen, 2012; Ettinger, Linzen, & Marantz, 2014) and can be assumed to correspond to a cost of storage, i.e., the minimum number of bits required to encode the word using the model.

The kind of information that is relevant to extract from the visual input during word recognition may depend on the task. Overall, we expect eye-movement measures to reflect at least to some extent more automatic processes than behavioral reaction times. In two experiments, we employ different ways of presenting the words to the participants during the measurement of their eye-movements: 1) standard visual lexical decision combined with eye-tracking, to enable direct comparisons to the previous lexical decision study by Virpioja et al., (2011b, 2018), and 2) a task in which the target words are presented embedded in rows of several unrelated letter strings, to better mimic eye-movement behavior in natural reading. In the latter task, the participants are to evaluate the lexicality of unrelated letter strings presented in the row (i.e., whether they were all real words or not). This is done in order to keep the main cognitive aspects of the second task as similar as possible to the lexical decision experiments. Additionally, by using unrelated words instead of sentences, we want to control for predictive spill-over effects from previous words, i.e., predicting upcoming words on the basis of sentence context (see, e.g., Hyönä, Vainio, & Laine, 2002). While the task is still essentially lexical decision, a behavioral response is not required on every item read, and the probability of observing a pseudoword is lower than in a standard lexical decision task. We assume that this aspect of the task reduces postlexical processes, such as demands to reanalyze the words, and puts more emphasis on primary lexical access processes in our measures. Thus, we ask to what extent the nature of the task affects the relative performance of the models, by comparing the standard visual lexical decision to a task that assumedly reduces the cost of reanalysis, check-up, and decision-making processes, which are likely to not be part of the most central aspects of word recognition in ecologically valid conditions.

Taken together, by using statistical models of morphology we study what kind of information is accessed during recognition of multimorphemic words. In particular, we are interested in the nature of the optimal units of processing (e.g., whether they are morpheme- or full-form-based) at different stages of word recognition and whether readers predict morphemes on the basis of previous ones. We additionally study to what extent particular task demands affect the kind of information used during online word recognition.

## Experiment 1

### Method

#### Participants

Twenty-four healthy volunteers (22 females; mean age 26.3 years; SD 5.6) participated in the lexical decision experiment. All were native speakers of Finnish with no diagnosed language disorders or neurological illnesses, and they were remunerated for their time. The study was approved by the Aalto University Research Ethics Committee.

#### Materials

The word stimuli were the same as those used in Virpioja et al., (2018) and consisted of 360 Finnish nouns with one or multiple (1-5) morphemes. In multimorphemic words the stem was accompanied by one or several inflectional, derivational, or possessive suffixes and/or clitic particles. The number of morphs was first calculated using the FINTWOL morphological analyzer (Lingsoft, Inc.) and further corrected by two native speakers of Finnish on the basis of linguistic assessment of derivational suffixes’ regularity and productivity, according to Karlsson (1983). The word materials had broad statistical distributions for several lexical parameters, permitting a correlational analysis for eye-tracking data. Three hundred words were randomly selected from the Morpho Challenge corpus (Kurimo, Creutz, & Varjokallio, 2008) including over 2.2 million word types and 44 million word tokens. This list was complemented with 60 additional randomly selected higher-frequency words because the random sample overemphasized low-frequency and bimorphemic words. For properties of the word stimuli with respect to statistical language models and descriptive statistics, see Table 1. Lemma frequency is the summative frequency of all the inflectional variants of a single stem (e.g., Baayen, Dijkstra, & Schreuder, 1997; Bertram, Baayen, & Schreuder, 2000, Taft, 1979) and assumed to affect the speed of accessing the stem when decomposing complex words. Morphological family size is the number of derivations and compounds where the noun occurs as a constituent (e.g., Bertram et al., 2000; del Prado, Bertram, Häikiö, Schreuder, & Baayen, 2004; Schreuder & Baayen, 1997).

**Table 1:** Properties of the stimulus words and cross-entropy values for the language models. For the models, the range and mean (SD) represent their self-information values

Predictor	Range	Mean (SD)	Cross-entropy
Number of letters	4-16	10.3 (2.8)	−
Number of morphs	1-5	2.8 (1.1)	−
Lemma frequency	1-54447	2215.3 (5218.6)	−
Morphological family size	1-5826	391.5 (791.4)	−
Word unigram	12.6-14.7	14.2 (0.6)	1.880
Morfessor *α* = 0.01	8.7-49.9	24.1 (6.6)	2.902
Morfessor *α* = 0.8	8.7-35.7	19.0 (4.5)	2.254
Morfessor *α* = 10.0	10.4-16.7	15.7 (1.1)	2.038
Morfessor CatMAP	8.2-36.5	17.3 (4.5)	2.019
Morph unigram	10.4-16.7	22.9 (5.9)	2.782
Morph bigram	8.6-29.4	15.7 (2.8)	1.944

In addition to the real words, 360 pseudoword items were included. They were produced with the help of a letter n-gram model, which estimates the probabilities of sequences of letters, and followed the phonotactic rules of Finnish. The length of the pseudowords was matched to the length of the real words, and they resembled the real words also in terms of their morphological structure. The lexical decision task included altogether 720 items.

#### Procedure

Participants were instructed to decide as quickly and as correctly as possible whether the letter string on the computer screen was a real word or not in Finnish and to press the corresponding button on a response pad. If a response had not been registered within 1500 ms of the presentation of the letter string, the letter string disappeared and a new fixation point would appear on the screen. Prior to the experiment, participants performed a practice block of 16 items (not included in the actual experiment) to familiarize themselves with the task. The eye-movements of the participants were registered using the EyeLink 1000 eye-tracking device (SR Research, Mississauga, Ontario, Canada) simultaneously to the lexical decision task. Recording of the eye-movements was performed on the right eye only and in the pupil-only mode with a sampling rate of 1000 Hz. The letter strings were presented in the middle of the screen in black Courier New font on a light gray background. The visual angle was 0.41 degrees. Prior to the task, calibration was performed using a nine-point grid that extended over the entire computer screen. Before the presentation of the letter string, a fixation cross was presented for 500 ms slightly to the left of each word. This position was chosen to better match its relative position in Experiment 2 in which the words were typically fixated after a saccade arriving from the left of the target word. A drift correction was performed after every third stimulus in the position of the fixation point. The items were divided into six blocks of equal length, the order of which was counterbalanced across participants, and there was a break after each block. Calibration was performed before each block.

#### Statistical models of interest

We investigated the performance of various statistical models of morphology in predicting eye-movement measures during recognition of a variety of morphologically simple and complex (inflected and derived) words. A summary of the included statistical models and their basic structure is provided in Table 2, and the descriptive statistics of the models in Table 1. A correlation table for word properties and statistical models is presented in Appendix A. Detailed model descriptions can be found in Appendix B. In the comparisons, we take into account the models’ cross-entropy, or text prediction accuracy, as this aspect of the models has been shown to affect models’ RT prediction ability in sentence processing (Fossum & Levy, 2012; Frank, 2009; Frank & Bod, 2011). Models with low cross-entropy are likely to work better than those with high cross-entropy. Empirical cross-entropy (see also Virpioja et al., 2018) is a standard evaluation measure for statistical language models in computational linguistics, and it estimates how unexpected a certain text corpus is with regard to the model trained by other text data (text prediction accuracy). Cross-entropy is the average self-information (surprisal) over all words in the text, here over our stimulus words.

**Table 2:** Evaluated language models categorized by their units of representation and the structure of the statistical model

Model units	Model structure	
	Context-independent	Context-dependent
Statistical morphs	Morfessor Baseline	Morfessor CatMAP
Linguistic morphs	Morph unigram	Morph bigram
Surface word forms	Word unigram	−

Our first aim was to study how the MDL-based optimization principle applied in Morfessor performs with the different eye-tracking measures. To address the question of optimal units of representation at different stages of word recognition, we study the basic version of Morfessor (“Morfessor Baseline”) and three of its variants that put differential emphasis on decomposition vs. full form representations via the manipulation of the hyper-parameter alpha. A model with a high alpha value segments little, whereas for a model with a small alpha value, the segmentations are closer to linguistically analyzed morphs. Similarly to Virpioja et al., (2018), we included models with alpha values of 0.01, 0.8, and 10.0 in the analysis. An alpha value of 0.01 was found to best correspond to a segmentation based on linguistic morphs, an alpha value of 10 is mostly based on full forms, and an alpha value of 0.8 which segments words at some morpheme boundaries and keeps some others unsegmented was found to perform best in the Virpioja et al., (2018) evaluation. The mean number of morphs per word for the stimulus words was 3.10 with the alpha value of 0.01, 1.74 with the alpha value of 0.8 and 1.00 with the alpha value of 10.0.

Our second aim was to investigate statistical models which predict upcoming morphological information on the basis of previously observed morphs. Whereas the Morfessor Baseline method assumes the morphs to appear independently of each other, the Morfessor CatMAP is a structured model, which assumes that words consist of prefixes, stems, and suffixes and categorical dependency between the units.

In addition, we studied the performance of other statistical models of morphology that also provide self-information estimates and that offer interesting comparison points. Such models include, e.g., the supervised morph n-gram models that utilize purely linguistically pre-segmented input in their estimates. They thus differ from Morfessor, which works in an unsupervised manner and builds a lexicon of statistical morphs (which may or may not correspond to linguistically defined morphemes) without this kind of given information. We included a morph unigram model and a bigram model in the analysis. Finally, we compared the models to a word unigram model based on surface frequencies, as a simple measure of whole-word processing.

In cases where model parameters needed to be tuned, the optimization was done using a lexical decision task dataset (Lehtonen, Cunillera, Rodríguez-Fornells, Hultén, Tuomainen, & Laine, 2007) used in Virpioja et al., (2011b). The stimulus words in that study were different from the ones included in the present experiments.

#### Data analyses

In Experiment 1, both eye-tracking and RT data were collected. Note that the RT data have been analyzed as part of the larger (Virpioja et al., 2018) lexical decision dataset. In the present study, we focused on eye-tracking data and also analyzed it with respect to additional statistical models (context-based models) than the RT data included in the Virpioja et al., (2018). To properly model the different random effects in the eye-tracking setup, the analyses were performed using the linear mixed effects modeling framework. The analyses were carried out using the R statistical computing platform and the “lme4” package (Bates, Mächler, Bolker, & Walker, 2015) for linear mixed modeling.

Our primary research question was how each language model alone, based on different kinds of representational units, can predict human word recognition. We thus included only particular setup-specific control predictors in the model, together with the language model of interest (see also Virpioja et al., 2018). In other words, we did not include psycholinguistic variables such as lemma frequency or morphological family size in the regression model, as the extent to which they account for the same variance as each of language models is likely to vary between the studied models, and including them would thus make interpretations difficult. The Akaike Information Criterion (AIC) (Akaike, 1974) value was used as a measure of the goodness of fit for the control predictors (smaller AIC values indicate better fit). The best fit was achieved by adding random intercepts for each subject and item. As a setup-specific control predictor, the words’ presentation order number was included in the model as a global estimate. In sum, each regression model included as predictors random effects for subject and item, one language model (e.g., Morfessor 0.8), and the setup-specific control predictor.

The goodness of fit for each language model was then evaluated by the decrease of the deviance of the regression model, i.e., improvement in comparison to the baseline regression model with only the control predictor and the random effects.

As an additional analysis, we included the word unigram model in the regression model together with each language model of interest and with the control predictors. This was done to see whether including models that allow morphological information improve the prediction of the eye-tracking measures beyond a model based on surface frequency.

##### Word length analyses (short vs. long words)

As previous eye-tracking studies have reported that short morphologically complex words are processed differently from long ones (see, e.g., Bertram & Hyönä, 2003), we ran further additional analyses regarding the relative performance of the models separately for short and long words. We divided our stimulus material to two groups on the basis of length (Bertram & Hyönä, 2003): our group of short words included items of eight letters or less, and the group of long words items of nine letters or more. We then ran similar regression analyses for short vs. long words as those performed for the whole set.

### Results

The items with erroneous responses and RTs with length of 3 SDs above or below the individual means were discarded from the data (1.6% of the data). We did not include the RTs in the analyses, as the RT and GD measures were highly correlated with a correlation value of 0.990. The measures would thus behave similarly also in the linear mixed modeling analyses. Three participants exceeded the preset error rate criterion of 15% and were excluded from the data. Also two stimulus words were excluded from the results because they shared the same stem. In cases of a single fixation on the word, GmF was entered as a missing value. In Experiment 1, 98% of the target word observations included more than one fixation. The descriptive statistics of the eye-tracking measures (FFD, GmF, GD) are presented in Tables 3 and 4. In this task, the highest correlations for global, late measures (GD and GmF) and language models were observed for Morfessor 0.8, followed by the morph bigram model. Of the language models, the morph unigram model showed the highest correlation with FFD.

**Table 3:** Descriptive statistics of the measures in Experiment 1. GD = Gaze duration; FFD = First fixation duration; GmF = GD minus FFD

Measure	Mean	SD
GD	769.3	246.3
FFD	244.2	102.6
GmF	537.4	260.95

**Table 4:** Correlations between background variables and the eye-tracking measures in Experiment 1. GD = Gaze duration; FFD = First fixation duration; GmF = GD minus FFD

Predictor	GD	FFD	GmF
Number of letters	0.528 (***)	− 0.621 (***)	0.612 (***)
Number of morphs	0.386 (***)	− 0.396 (***)	0.433 (***)
Lemma frequency	− 0.309 (***)	− 0.006	− 0.293 (***)
Morphological family size	− 0.267 (***)	− 0.072	− 0.238 (***)
Word unigram	0.580 (***)	− 0.222 (***)	0.573 (***)
Morfessor *α*= 0.01	0.510 (***)	− 0.370 (***)	0.544 (***)
Morfessor *α*= 0.8	0.613 (***)	− 0.384 (***)	0.639 (***)
Morfessor *α*= 10.0	0.589 (***)	− 0.264 (***)	0.599 (***)
Morfessor CatMAP	0.582 (***)	− 0.317 (***)	0.588 (***)
Morph unigram	0.499 (***)	− 0.407 (***)	0.542 (***)
Morph bigram	0.594 (***)	− 0.340 (***)	0.618 (***)

*** *p* <.001. ** *p* <.01. * *p* <.05

The decreases in the regression model deviance and the corresponding *p* values are presented in Table 5. Morfessor Baseline variant with an alpha value of 0.8 predicted GDs best in the present data. It outperformed the morph bigram model, Morfessor CatMAP, Morfessor Baseline with an alpha of 10, and the word unigram model, despite the fact that all of these had a more favorable cross-entropy.

**Table 5:** AIC values, decrease in the regression model deviance, and *p* values for the primary analysis of Experiment 1. AIC = Akaike Information Criterion; GD = Gaze duration; FFD = First fixation duration; GmF = GD minus FFD

Predictor	GD			FFD			GmF		
	AIC	Δ	*p* value	AIC	Δ	*p* value	AIC	Δ	*p* value
Word unigram	− 1092	144.136	3.318e-33 (***)	7804	19.079	1.254e-05 (***)	7426	144.629	2.588e-33 (***)
Morfessor *α*= 0.01	− 1059	111.263	5.182e-26 (***)	7772	50.154	1.421e-12 (***)	7436	134.121	5.139e-31 (***)
Morfessor *α*= 0.8	− 1121	172.782	1.826e-39 (***)	7766	56.178	6.621e-14 (***)	7376	194.143	3.963e-44 (***)
Morfessor *α*= 10.0	− 1098	150.151	1.607e-34 (***)	7796	27.084	1.948e-07 (***)	7410	160.685	8.015e-37 (***)
Morfessor CatMAP	− 1101	152.696	4.464e-35 (***)	7786	36.535	1.499e-09 (***)	7415	155.759	9.558e-36 (***)
Morph unigram	− 1056	107.965	2.735e-25 (***)	7762	60.721	6.576e-15 (***)	7438	132.458	1.188e-30 (***)
Morph bigram	− 1106	157.895	3.262e-36 (***)	7781	41.737	1.044e-10 (***)	7394	176.908	2.294e-40 (***)

*** *p* <.001. ** *p* <.01. * *p*<.05

**Fig. 1:**
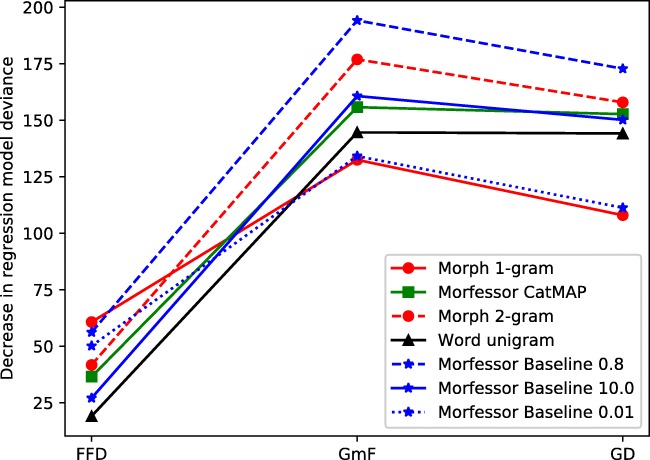
Experiment 1. Decrease in mixed model deviance for the different measures and models. A higher value indicates better fit. GD = Gaze duration; FFD = First fixation duration; GmF = GD minus FFD

With respect to the question of the time-course of these effects, we focused on FFD and GmF measures (see Fig. 1), assumedly reflecting early and late stages of processing, respectively. We also plotted the measures as a function of the cross-entropy of the model (see Fig. 3 in Appendix C). All language models provided significant improvements to the baseline regression models. In the FFDs, the best-performing models were the morph unigram model, Morfessor Baseline 0.8, and Morfessor Baseline 0.01, followed by the morph bigram model and Morfessor CatMAP. The whole-word based measures Morfessor 10 and the word unigram model did not predict FFDs quite as well. In the GmFs, the best predictor was Morfessor 0.8, followed by the morph bigram model, Morfessor 10, and CatMAP. In sum, in the early measure the decomposing models fared relatively better than whole-word-based or context-predicting models, despite their higher cross-entropies. In the later phase, however, models that allow also whole-word processing or predict following morphs on the basis of previous ones were relatively better predictors than the strongly decomposing models.

The additional analyses including word unigram in the regression model together with each language model showed that each of the language models improved the prediction of all of the dependent measures beyond the word unigram model (see Table 14 in the Appendix D). In other words, models that allow, at least to some extent, morphological information to be utilized account for variance in the data that is not explained merely by full-form aspects of processing.

#### Analyses for short and long words

The descriptive correlations for short and long words are presented in Appendix E. The regression results for the long words are presented in Table 6 and for the short words in Table 7. The regression analyses performed separately for short and long words showed that processing varied somewhat between them. For short words, GDs were best predicted by Morfessor Baseline 10, the word unigram model and the morph bigram model. In long words, however, the best predictors of GDs were Morfessor Baseline 0.8 and Morfessor CatMAP. In the early measure FFD, Morfessor 0.8 performed well in both short and long words. In short words, the morph bigram model also performed well, perhaps partly due to its favorable cross-entropy. For long words, the morph unigram model performed best together with Morfessor 0.8. In the late measure GmF, Morfessor Baseline 10, the morph bigram model, and Morfessor 0.8 were the best predictors for short words. In long words, Morfessor Baseline 0.8 and Morfessor CatMAP fared best in GmF, instead of a fully whole-word-based measure, suggesting a somewhat stronger involvement of morpheme-based processing for long than short words.

**Table 6:** AIC values, decrease in the regression model deviance, and *p* values for the long words in Experiment 1. AIC = Akaike Information Criterion; GD = Gaze duration; FFD = First fixation duration; GmF = GD minus FFD

Predictor	GD			FFD			GmF		
	AIC	Δ	*p* value	AIC	Δ	*p* value	AIC	Δ	*p* value
Word unigram	− 1033	70.023	5.861e-17 (***)	4422	4.396	3.602e-02 (*)	4589	73.979	7.896e-18 (***)
Morfessor *α*= 0.01	− 1009	45.106	1.867e-11 (***)	4421	5.504	1.898e-02 (*)	4613	50.673	1.091e-12 (***)
Morfessor *α*= 0.8	− 1045	81.252	1.987e-19 (***)	4417	9.142	2.498e-03 (**)	4576	87.359	9.050e-21 (***)
Morfessor *α*= 10.0	− 1031	67.082	2.604e-16 (***)	4421	5.316	2.114e-02 (*)	4594	69.674	6.998e-17 (***)
Morfessor CatMAP	− 1046	83.056	7.978e-20 (***)	4419	6.781	9.215e-03 (**)	4583	80.568	2.809e-19 (***)
Morph unigram	− 999	36.038	1.936e-09 (***)	4417	9.204	2.415e-03 (**)	4618	44.946	2.026e-11 (***)
Morph bigram	− 1037	73.281	1.124e-17 (***)	4421	5.174	2.293e-02 (*)	4586	77.256	1.502e-18 (***)

*** *p* <.001. ** *p* <.01. * *p*<.05

**Table 7:** AIC values, decrease in the regression model deviance and *p* values for the short words in Experiment 1. AIC = Akaike Information Criterion; GD = Gaze duration; FFD = First fixation duration; GmF = GD minus FFD

Predictor	GD			FFD			GmF		
	AIC	Δ	*p* value	AIC	Δ	*p* value	AIC	Δ	*p* value
Word unigram	− 353	47.970	4.327e-12 (***)	3029	0.266	6.059e-01	2512	43.049	5.339e-11 (***)
Morfessor *α*= 0.01	− 320	14.990	1.081e-04 (***)	3026	3.012	8.264e-02	2535	20.010	7.703e-06 (***)
Morfessor *α*= 0.8	− 345	40.024	2.509e-10 (***)	3023	6.108	1.345e-02 (*)	2509	45.855	1.273e-11 (***)
Morfessor *α*= 10.0	− 355	49.628	1.859e-12 (***)	3028	1.134	2.869e-01	2501	54.283	1.736e-13 (***)
Morfessor CatMAP	− 336	31.361	2.142e-08 (***)	3026	2.468	1.162e-01	2522	33.098	8.763e-09 (***)
Morph unigram	− 325	19.661	9.247e-06 (***)	3027	2.051	1.521e-01	2536	18.878	1.393e-05 (***)
Morph bigram	− 347	41.907	9.570e-11 (***)	3024	4.407	3.579e-02 (*)	2502	52.951	3.420e-13 (***)

*** *p* <.001. ** *p* <.01. * *p*<.05

### Discussion

In this lexical decision experiment that utilized eye-tracking, we compared the relative performance of various statistical language models and investigated temporal aspects of recognition of morphologically complex words. In the eye-tracking measures, we found that overall, a variant of Morfessor Baseline (with an alpha value of 0.8) which decomposes words at some morpheme boundaries and leaves others unsegmented, showed very good performance, especially with global and late reading measures (GD and GmF) but also in FFDs assumedly reflecting earlier stages of processing. This was shown both in the correlations and regression analyses and is in line with the RT results of Virpioja et al., (2018). A Morfessor Baseline variant 0.01 which segments most of the morpheme boundaries performed relatively better in the early measure than the late ones. In contrast, the whole-word-based models, the word unigram model and Morfessor 10, were not particularly good predictors of this early measure; however, they fared relatively better in the late measures.

Overall, the early measures were best predicted by models that assume that all morphs occur independently (Morfessor Baseline, morph unigram model) and that segment words into morphemes. In the late measures, however, the models that predict morphs on the basis of previous ones and those that also allow whole words to be stored performed relatively better. This suggests that morpheme-based information is recruited early in the course of word recognition, largely in line with early decomposition accounts (e.g., Rastle and Davis, 2008). However, the good performance of Morfessor Baseline 0.8 that does not segment all morpheme boundaries suggests that some full-form information is coded at this stage as well. The later stages, in turn, seem to utilize several different sources of information: At that level, particularly predictions made on the basis of previous morphemes may be utilized (as reflected in the performance of relatively good performance of the morph bigram model and CatMAP). Models based only on decomposed, independent morphemes (Morfessor 0.01 and the morph unigram model) did not perform particularly well in the late measure, suggesting that some information about the whole word is used at this point, either via whole-word representations or via online integration of morphemes into semantically coherent concepts. Decision-making processes and analyzing the correctness of the morpheme combination can also be assumed to be part of the later measures in this single-word lexical decision task.

Word length modulated these effects to some extent. Morfessor Baseline with an alpha value of 0.8 and its optimization between decomposition and full-form processing showed good performance in the early measure in both short and long words. In general, morpheme-based models performed better at this early stage than full-form-based ones in both short and long words, supporting the view that morphological information is accessed early in many words. At later stages of word recognition, there was evidence of whole-word processing, particularly in short words, whereas in long words an optimized solution between decomposition and full-form processing seems to function best. At later stages, processing also involves predicting upcoming morphemes from previous morphemes, both in short and long words. In compound words embedded in sentences, Bertram and Hyönä (2003) observed whole-word frequency effects for short words in early and late processing measures but for long words only in late measures. Here, in multimorphemic derived and inflected words, we found that an optimized solution for decomposing words at some morpheme boundaries early and keeping others unsegmented works for both short and long words. In long words, a stronger emphasis is placed on decomposition, both in early and late measures.

The results from this combined eye-tracking and lexical decision experiment suggest that the independent predictive power of both Morfessor Baseline 0.8 and the word unigram model based on whole words in the Virpioja et al., (2018) study reflect somewhat different stages of word recognition. The good performance of Morfessor Baseline 0.8 seems to reflect both early and late word recognition processes, but the whole-word measures primarily the later stages. The later stages in the lexical decision task can be assumed to incorporate several processes: semantic and syntactic integration of morphological constituents to a unified whole as well as postlexical processes such as checking the correctness of the combination and decision making processes. In Experiment 2, we aimed to reduce the emphasis on such postlexical processes in the task. This was done in order to shed light on the more specific source of the whole-word effects and to investigate processing of multimorphemic words by assumedly taking one step towards more natural reading.

## Experiment 2

### Method

Lexical decision, while previously also studied in an eye-tracking context, (e.g., Kuperman, Drieghe, Keuleers, & Brysbaert, 2013), is a task that entails presenting single words one at a time on the screen. In Experiment 2, in turn, words were presented in sequences on the screen. In this task, the participants were to evaluate the correctness of each word, but not to give a response to every single word. Furthermore, most/all of the items in a row were real words, making the task in this respect closer to natural reading than the standard visual lexical decision. We argue that in a task in which the probability of observing a pseudoword is relatively low for each item to be read (only half of the rows included one pseudoword), decision and/or reanalysis processes are not as costly as in the standard lexical decision task. Presentation of words in rows can also be assumed to lead to increasingly ecologically valid eye-movement behavior. At the same time, the aim was to reduce predictive spill-over effects from previous words that can be assumed to be more pronounced in sentences than when using unrelated words. The aim was to see to what extent the kind of morphological processing observed in the single-word visual lexical decision task is task-specific.

#### Participants

Twenty-six healthy volunteers (22 females; mean age, 22.6; SD 2.7) participated in the experiment. The inclusion criteria were the same as those in Experiment 1, but none of the participants of Experiment 2 had taken part in that experiment.

#### Materials

The target word materials were the same as those used in Experiment 1. In this experiment, the target words were embedded in rows of unrelated nouns. All rows included seven words, and the target words always occupied the third and fifth position in the row while the rest of the items were filler words. The unrelated filler nouns in the row were randomly selected from the Morpho Challenge corpus with similar criteria as those used in the selection of target words in terms of their length and morphological structure, i.e., they could be mono- or multimorphemic nouns. Altogether 180 rows that included these target words were presented to the participants. In order to prevent possible order effects within the row due to particular items occurring before or after one another, four pseudorandomized lists were created in which the words were shuffled. In other words, the position and row in which each word (both targets and fillers) was located was pseudorandomly varied between these lists, but such that there was a target word always in the third and fifth position. Each participant was assigned to one of these lists. Additionally, there were 180 rows which included one pseudoword. The pseudowords used in this experiment were randomly selected from the pseudowords used in Experiment 1. This pseudoword item could take any place in the row, and the frequency of a pseudoword’s occurrence in each of these different positions was balanced to the extent possible.

#### Procedure

The eye-movement recordings were performed using the same device and similar settings as those of Experiment 1. A fixation cross was presented on the left side of the screen slightly to the left of the presentation location of the first word. Participants were instructed to read the word rows silently at their own pace. Occasionally after the row, a question was presented on the screen asking whether the previous row included a pseudoword. The question appeared randomly but on average after every third row. Participants were to press a button indicating whether the previous row had included a pseudoword or not. After having finished reading each row, participants pressed a button signaling they wanted to move on. The maximum time to read a row was 10 s. The order of the rows was randomized, and the rows were presented in nine blocks of 40 rows each. There was a break between the blocks, and after each break a new calibration was performed. Prior to the experiment, participants performed a practice block of 15 rows (not included in the actual experiment) to familiarize themselves with the task.

#### Data analyses

Similarly to Experiment 1, the data analyses were carried out in a linear mixed modeling framework using the “lme4” package (Bates et al., 2015) for the R statistical computing platform.

We included a number of setup-specific control predictors in the regression model. We evaluated different ways for modeling these control predictors and used the AIC value as a measure of the goodness of fit for the resulting model. Random intercepts were added for each subject, item, and list of words presented to the subject. Presentation order and the eye-movement launch site were included in the model as global estimates. The launch site is defined as the distance between the position of the last fixation in the previous interest area (word preceding the stimulus word) and the left border of the current interest area (stimulus word). The position of the analyzed word in the shown row of words (third or fifth word) was modeled as a per-subject slope. In sum, each regression model included as predictors random effects for subject, item and list, one language model (e.g. Morfessor 0.8), and the setup-specific control predictors (launch site, presentation order of the items, per-subject-slope for word’s position in the row).

Similarly to Experiment 1, in the additional analyses we included the word unigram model in the regression model together with each language model of interest and with the control predictors. This was done to see whether including models that allow morphological information improve the prediction of the eye-tracking measures beyond surface frequency. Furthermore, we analyzed the data regarding the relative performance of the models separately for short and long words.

### Results

In Experiment 2, we focused our analyses on first-pass reading, to avoid the strategic re-reading and check-up processes that have a larger role after the participant has read the word once. The dependent measures of interest were again GD, FFD, and GD minus FFD: GmF. The items with GDs with duration of 3 SDs above or below the individual means were discarded from the data (3.0% of the data). In cases of a single fixation on a word, GmF was entered as a missing value. in Experiment 2, 63% of the target word observations included more than one fixation. The descriptive statistics of the eye-tracking measures (FFD, GmF, GD) are presented in Tables 8 and 9. The highest correlations between GD and GmF measures and language models were observed for Morfessor 0.8, followed by the morph unigram model. For FFD, the morph unigram model, in turn, showed the highest correlation, followed by the highly segmenting Morfessor 0.01.

**Table 8:** Descriptive statistics of the measures in Experiment 2. GD = Gaze duration; FFD = First fixation duration; GmF = First run GD minus FFD

Measure	Mean	SD
First run GD	610.7	349.9
FFD	306.4	108.8
GmF	483.8	321.6

**Table 9:** Correlations between background variables and the eye-tracking measures in Experiment 2. GD = First run gaze duration; FFD = First fixation duration; GmF = GD minus FFD

Predictor	GD	FFD	GmF
Number of letters	0.829 (***)	− 0.495 (***)	0.638 (***)
Number of morphs	0.574 (***)	− 0.304 (***)	0.463 (***)
Lemma Frequency	− 0.146 (**)	− 0.160 (**)	− 0.067
Morphological family size	− 0.116 (*)	− 0.153 (**)	− 0.047
Word unigram	0.504 (***)	− 0.030	0.393 (***)
Morfessor *α* = 0.01	0.598 (***)	− 0.287 (***)	0.428 (***)
Morfessor *α* = 0.8	0.645 (***)	− 0.165 (**)	0.532 (***)
Morfessor *α* = 10.0	0.535 (***)	− 0.025	0.404 (***)
Morfessor CatMAP	0.574 (***)	− 0.115 (*)	0.489 (***)
Morph unigram	0.630 (***)	− 0.316 (***)	0.516 (***)
Morph bigram	0.601 (***)	− 0.153 (**)	0.493 (***)

*** *p* <.001. ** *p* <.01. * *p* <.05

**Fig. 2:**
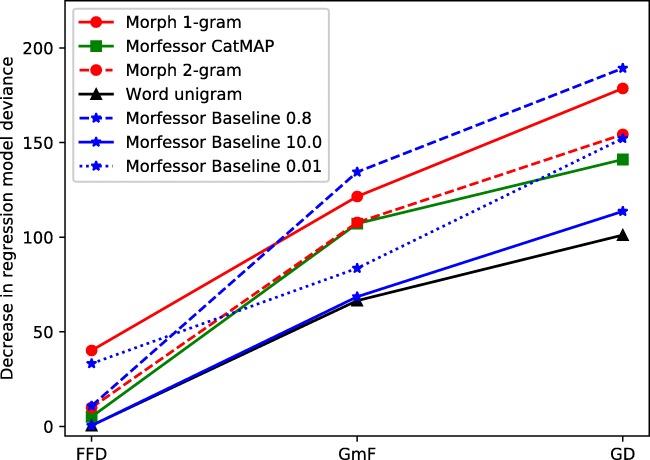
Experiment 2. Decrease in mixed model deviance for the different measures and models. Higher value indicates better fit. GD = First run gaze duration; FFD = First fixation duration; GmF = GD minus FFD

**Fig. 3:**
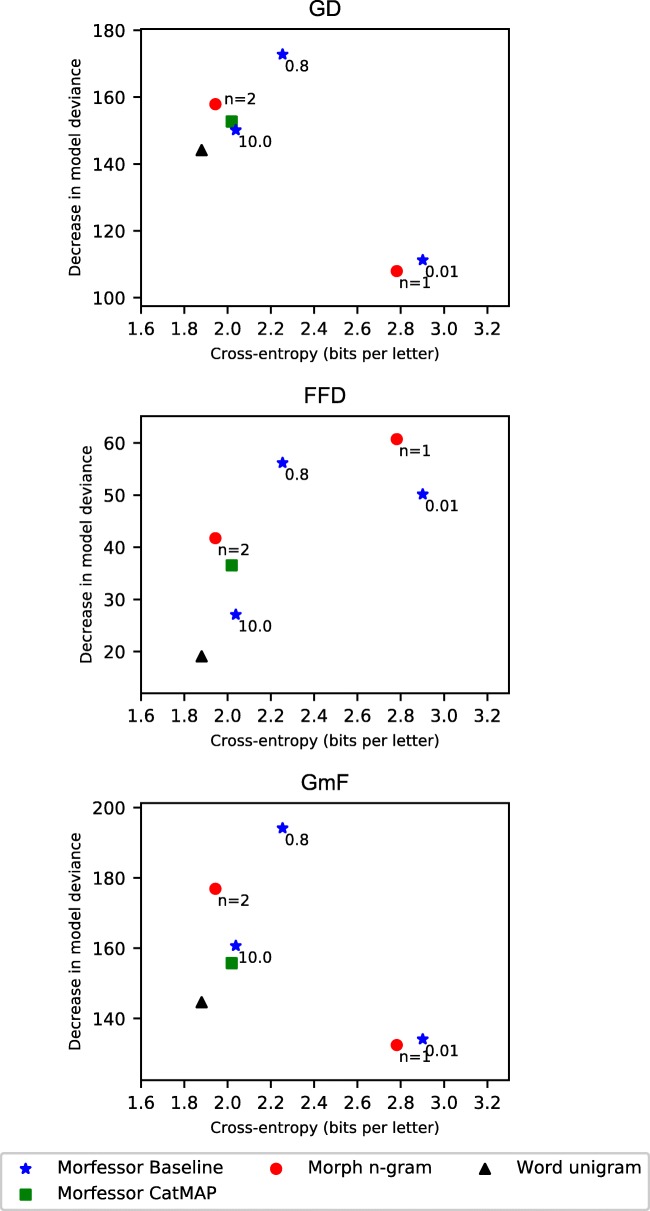
Experiment 1. Decrease in mixed model deviance as a function of the cross-entropy of the model. GD = Gaze duration; FFD = First fixation duration; GmF = GD minus FFD

**Fig. 4:**
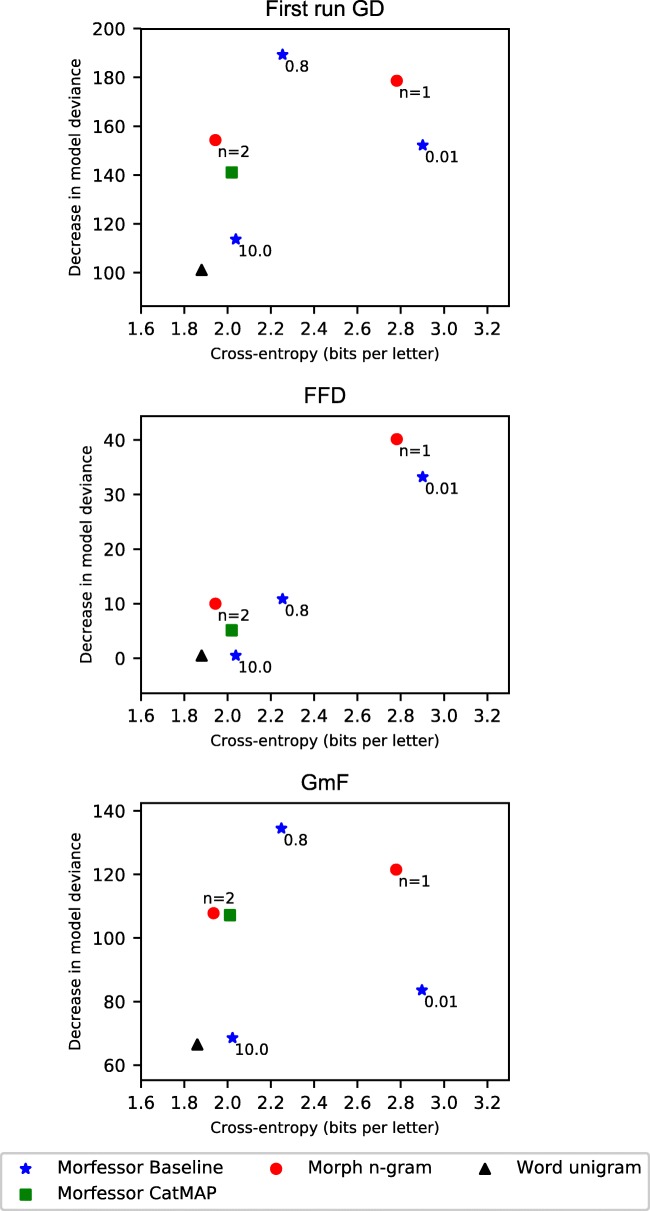
Experiment 2. Decrease in mixed model deviance as a function of the cross-entropy of the model. GD = Gaze duration; FFD = First fixation duration; GmF = First run GD minus FFD

The decreases in the regression model deviance and the corresponding p values are presented in Table 10. All the studied language models improved the baseline regression model (Fig. 2). The measures were also plotted as a function of the cross-entropy of the model (see Fig. 4 in the Appendix C). With regard to GD, the best improvements to the baseline regression model were provided by Morfessor Baseline with an alpha value of 0.8, the morph unigram model, the morph bigram model, and Morfessor Baseline 0.01, followed by CatMAP. The whole-word models Morfessor 10 and the word unigram models were the lowest in this ranking.

**Table 10:** AIC values, decrease in the regression model deviance and *p* values for the primary analysis of Experiment 2. AIC = Akaike Information Criterion; GD = Gaze duration; FFD = First fixation duration; GmF = First run GD minus FFD

Predictor	First run GD			FFD			GmF		
	AIC	Δ	*p* value	AIC	Δ	*p* value	AIC	Δ	*p* value
Word unigram	8176	101.145	8.550e-24 (***)	3603	0.492	4.832e-01	6855	66.497	3.505e-16 (***)
Morfessor *α* = 0.01	8125	152.194	5.746e-35 (***)	3570	33.220	8.231e-09 (***)	6838	83.582	6.112e-20 (***)
Morfessor *α* = 0.8	8088	189.294	4.533e-43 (***)	3593	10.851	9.873e-04 (***)	6787	134.461	4.331e-31 (***)
Morfessor *α* = 10.0	8163	113.669	1.540e-26 (***)	3603	0.478	4.895e-01	6853	68.553	1.235e-16 (***)
Morfessor CatMAP	8136	141.080	1.545e-32 (***)	3598	5.119	2.367e-02 (*)	6814	107.197	4.031e-25 (***)
Morph unigram	8098	178.618	9.709e-41 (***)	3563	40.155	2.346e-10 (***)	6800	121.509	2.957e-28 (***)
Morph bigram	8123	154.322	1.969e-35 (***)	3593	9.994	1.571e-03 (**)	6813	107.797	2.978e-25 (***)

*** *p* <.001. ** *p* <.01. * *p*<.05

In the early measure FFD, the strongly morpheme-based models morph unigram model and Morfessor Baseline 0.01 provided the best improvements to the baseline regression model, followed by Morfessor Baseline 0.8, the morph bigram model, and CatMAP. Morfessor 10 and the word unigram model did not significantly improve the baseline model. In the late measure GmF, Morfessor 0.8 provided the best improvements, followed by the morph unigram and bigram models and CatMAP. Morfessor 0.01, Morfessor 10, and the word unigram model, while providing significant improvements, were not as good predictors of GmF as the other models.

The additional analyses including word unigram in the regression model together with each language model showed that almost all of the language models improved the prediction of the dependent measures beyond the word unigram model, the only exception being Morfessor 10 in the FFD measure (see Table 17 in the Appendix F). This shows that models that at least to some extent incorporate morphological information explain variance in the data beyond a model based on surface frequencies.

#### Analyses for short and long words

The descriptive correlations between eye-tracking measures and short and long words are presented in Appendix G and the regression results in Tables 11 and 12. For short words, the whole-word models dominated: the word unigram model and Morfessor Baseline 10 were the best predictors of the global measure GD, but also of the early measure FFD. In the FFD, these whole-word models were followed by Morfessor 0.8 and CatMAP. For GmF, the word unigram model was also the best predictor, but in this measure it was followed by Morfessor CatMAP, Morfessor 10 and 0.8. In contrast, in long words, there was more emphasis on morphemes, both statistical and linguistic ones: In the FFD, the morph unigram model based on linguistic morphemes was the best predictor, followed by Morfessor 0.01. In GD and GmF, Morfessor Baseline 0.8, in turn, was clearly the best predictor, followed by the morph bigram model and Morfessor CatMAP.

**Table 11:** AIC values, decrease in the regression model deviance and *p* values for the long words in Experiment 2. AIC = Akaike Information Criterion; GD = Gaze duration; FFD = First fixation duration; GmF = First run GD minus FFD

Predictor	First run GD			FFD			GmF		
	AIC	Δ	*p* value	AIC	Δ	*p* value	AIC	Δ	*p* value
Word unigram	5846	47.044	6.942e-12 (***)	2002	0.087	7.677e-01	5735	47.707	4.948e-12 (***)
Morfessor *α* = 0.01	5845	48.152	3.944e-12 (***)	1995	6.696	9.663e-03 (**)	5746	36.127	1.849e-09 (***)
Morfessor *α* = 0.8	5812	81.393	1.850e-19 (***)	2001	0.356	5.507e-01	5703	79.279	5.393e-19 (***)
Morfessor *α* = 10.0	5842	51.008	9.200e-13 (***)	2002	0.001	9.733e-01	5733	49.421	2.066e-12 (***)
Morfessor CatMAP	5831	62.245	3.032e-15 (***)	2002	0.017	8.973e-01	5719	63.522	1.586e-15 (***)
Morph unigram	5839	54.358	1.671e-13 (***)	1991	10.422	1.245e-03 (**)	5728	54.282	1.737e-13 (***)
Morph bigram	5828	65.143	6.966e-16 (***)	2001	0.493	4.825e-01	5716	66.093	4.301e-16 (***)

*** *p* <.001. ** *p* <.01. * *p*<.05

**Table 12:** AIC values, decrease in the regression model deviance and *p* values for the short words in Experiment 2. AIC = Akaike Information Criterion; GD = Gaze duration; FFD = First fixation duration; GmF = First run GD minus FFD

Predictor	First run GD			FFD			GmF		
	AIC	Δ	*p* value	AIC	Δ	*p* value	AIC	Δ	*p* value
Word unigram	2085	36.138	1.838e-09 (***)	1404	15.803	7.029e-05 (***)	1043	5.628	1.768e-02 (*)
Morfessor *α* = 0.01	2106	14.817	1.185e-04 (***)	1415	5.278	2.160e-02 (*)	1048	0.176	6.752e-01
Morfessor *α* = 0.8	2091	30.606	3.161e-08 (***)	1407	12.902	3.283e-04 (***)	1045	3.025	8.200e-02
Morfessor *α* = 10.0	2086	35.093	3.144e-09 (***)	1404	16.280	5.464e-05 (***)	1045	3.020	8.226e-02
Morfessor CatMAP	2091	30.282	3.735e-08 (***)	1408	12.181	4.828e-04 (***)	1045	3.390	6.560e-02
Morph unigram	2102	19.313	1.109e-05 (***)	1410	10.095	1.487e-03 (**)	1047	1.497	2.211e-01
Morph bigram	2091	29.836	4.701e-08 (***)	1412	8.015	4.639e-03 (**)	1047	1.461	2.268e-01

*** *p* <.001. ** *p* <.01. * *p*<.05

### Discussion

Experiment 2 utilized a task which required assessing the lexicality of letter strings in a similar way to lexical decision; however, in this task nouns were presented in sequences, i.e., as rows of text on the screen, in order to take a step towards mimicking eye-movements during natural reading. We also assumed that this task in which the probability of seeing a real word was higher than in lexical decision would diminish the role of postlexical processes related to reanalysis and decision-making. In fact, the stimulus words elicited a greater amount of single fixations in this word sequence task than in the lexical decision task of Experiment 1, suggesting a generally smaller role for reanalysis processes in the measures obtained in this task.

Overall, the results for the global measure of GD again showed very good performance of Morfessor Baseline with an alpha value of 0.8, which segments some morpheme boundaries while not others. The early stages were, however, even better predicted by the morph unigram model that is based on linguistically defined morphemes, as well as Morfessor Baseline 0.01 that produces segmentations relatively close to linguistic morphemes. These findings provide evidence for the view that morphologically decomposed representations are accessed at early stages of word recognition (e.g., Rastle & Davis, 2008).

In the late measure GmF, both the models based on statistical morphs (that also allow full-form representations: Morfessor Baseline 0.8 and CatMAP) as well as those based on linguistic morphemes fared well. Overall, the context-based models (CatMAP and the morph bigram model) improved their ranking in the late measure in comparison to the early measure FFD. These findings suggest that later stages of word recognition utilize largely morpheme-based representations but also those that the MDL principle finds optimal, i.e., sometimes keeping constituent boundaries unsegmented. In addition to morphological information, predictive information of previous morphemes is further utilized. Interestingly, the fully whole-word-based models Morfessor 10 and the word unigram model did not perform well in comparison to the other models even in the late eye-tracking measures when considering the full set of stimulus words.

The lexical decision task used in Experiment 1 is the most commonly used task for investigating morphological processing. It can be assumed to emphasize processes related to reanalysis of the word and decision-making to a greater extent than the present task. It is therefore interesting to note that the predictive power of whole word based models in Experiment 1 might primarily stem from these kinds of postlexical processes, and it thus seems that full-form variables in fact play a smaller role in increasingly natural reading or word recognition where careful consideration of lexicality of each item is usually not that central.

The picture, however, looks somewhat different when comparing the relative performance of the models in short words vs. long words separately. For short words, full-form models predicted both early and late measures, even in this task. A word that can be seen in one fixation can be recognized via a full form if such a representation exists for it. In longer words, early processing seems to be governed by linguistically defined morphemes, and also at later stages there is more emphasis on models based on morphemes or an optimized combination of morphemes and full-forms. These results are consistent with findings of Bertram and Hyönä (2003) who used compound words, finding early as well as late full-form effects for short compounds. Our present results from Experiment 1 are also more in line with the Bertram and Hyönä (2003) findings than the results from Experiment 1, likely because the word recognition task of Experiment 2 was somewhat more similar to the sentence reading task of Bertram and Hyönä (2003).

Taken together, these results suggest that under somewhat more naturalistic word recognition conditions, morpheme-based information is accessed early in the processing, whereas at later stages, the processing system takes into account different kinds of information: It utilizes predictions made on the basis of previous morphemes as well as information that is coded in the MDL principle of Morfessor 0.8, combining decomposed representations with some unsegmented morpheme combinations. This is the case at least in longer words. For short words, full-form processing seems to dominate.

## General discussion

We studied how statistical models of morphology that are built on different kinds of representational units perform in predicting human word recognition. By using eye-tracking, we aimed to shed light on the kinds of processing units accessed at early vs. late stages of recognition of morphologically complex words. In combination with the eye-movement registrations, we used two tasks, a standard visual lexical decision task (Experiment 1) and a variant of lexical decision in which the target words were presented in word sequences, i.e., surrounded by other words in rows, to take a step towards mimicking eye-movement behavior in natural reading (Experiment 2). The use of these two tasks allowed us to disentangle the contribution of task-related effects in morphological processing, as we assumed the latter task to put less emphasis on postlexical reanalysis and decision processes. Similarly to Virpioja et al., (2018), our focus was on unsupervised statistical models that produce self-information estimates, in particular on the Morfessor model family (Creutz and Lagus, 2002, 2007). We compared them to similar simple supervised models based on linguistic morphs (morph n-gram models), as well as a whole-word-based word unigram model. In addition to the time-course of these effects, we also investigated another novel aspect in processing of multimorphemic words, prediction of upcoming morphs on the basis of previous ones.

As expected based on Virpioja et al., (2018), the lexical decision task of Experiment 1 showed that the global measure GD was best predicted by Morfessor Baseline 0.8 which segments words at some morpheme boundaries while not at others. Full-form models as well as models that take into account morpheme context, i.e., predicted morphemes on the basis of previous ones, also performed well. Also in Experiment 2, the global GDs were best predicted by Morfessor Baseline 0.8. In this word recognition task, a relatively stronger involvement of linguistically defined morphemes was observed than in the lexical decision task, as shown by the good performance of the morph unigram and bigram models and Morfessor Baseline 0.01. In other words, in this assumedly more ecologically valid task, full-form processing is not as prominent in this global measure as in the single word recognition task in which reanalysis and decision processes are more central.

Morfessor models are based on the MDL principle and work to optimize the costs related to storage and computation of complex words, and different model variants differentially emphasize these constraints. Virpioja et al., (2018) analyzed systematically the segmentations produced by Morfessor Baseline instances 0.8 and 0.01. This analysis showed that while outputs of Morfessor 0.01 are rather close to linguistically defined morphemes, Morfessor 0.8 keeps many affixes unsegmented. In the analysis, clitic particles were left distinct, but morpheme boundaries at derivational suffixes were most often left unsegmented. Similarly, Morfessor 0.8 left two-thirds of bimorphemic stem + inflectional suffix combinations unsegmented. The good performance of Morfessor 0.8 in the present study thus provides further support for the parallel dual-route framework in which both decomposed and holistic representations can be utilized for morphologically complex words .

Eye-tracking allowed us to look more closely into the temporal stages of word recognition that the good performance of the models in each task stems from. Early stages of word recognition were well predicted by models based on linguistically defined morphemes as well as Morfessor Baseline 0.8 which includes a combination of segmented and non-segmented morpheme boundaries. This observation was made both in the lexical decision task of Experiment 1 and in the word sequence task of Experiment 2. In the latter task, however, a stronger emphasis for linguistic morphemes was found. This finding is in line with cognitive models that assume that words are segmented into their morphological constituents early (e.g., Rastle and Davis, 2008; Taft, 1979, 2004). Nevertheless, we also found evidence supporting models that assume that units larger than morphemes can sometimes be accessed at this early stage (Frauenfelder & Schreuder, 1992; Diependaele, Sandra, & Grainger, 2009; Schreuder & Baayen, 1995).

The later stages of word recognition can be assumed to incorporate several processes: semantic and syntactic access and integration of morphological constituents to a unified whole. A lexical decision task additionally incorporates licensing (see Schreuder & Baayen, 1995) and decision processes to this cascade. Whereas our results for the early stages were fairly similar irrespective of the task, measures reflecting later stages of word recognition differed for the two tasks of the present experiments. The single-word lexical decision results of Experiment 1 would suggest that later-stage processing of complex words takes place by accessing full-form representations, in addition to utilizing morpheme-based and predictive information from previous morphemes. Assuming that the task of Experiment 2 reduced the role of postlexical processes, the present study suggests that the particularly good performance of purely full-form models in the late measures of the single-word lexical decision task largely stems from postlexical decision- and reanalysis-related processes. An interesting further experiment to corroborate this interpretation would be one in which the target words were presented embedded in natural sentences. Based on the current results, we would expect to observe that the later stages of core word reading processes utilize morpheme-based and predictive information to a significant extent, while processing some constituent boundaries as unsegmented as well, especially in shorter words.

Virpioja et al., (2018) showed the best performance for RT prediction when including both Morfessor 0.8 and the word unigram model (based on whole-word frequency) in the regression analysis. The present results from our combined eye-tracking and lexical decision experiment (Experiment 1) indicate that the distinct prediction abilities for these two models reflect partly different stages of word recognition. The good performance of Morfessor 0.8 in the Virpioja et al., (2018) study seems to reflect both early and late word recognition processes, but the whole-word measures primarily the later stages. This observation is in line with previous proposals associating surface frequency to the later stage of morphological processing (e.g., Taft, 2004). However, the present comparison to Experiment 2, in which full-form measures performed overall poorly, suggests that these measures are observed to be influential particularly when analysis and decision of the correctness of the word form (licensing) are central rather than in more natural word recognition conditions. An exception to this are short complex words that largely seem to be processed as full forms even in a task in which most words are correct and the role of reanalysis processes is likely to be smaller. In fact, word length seems to play a notable role in determining the most optimal processing route for complex words. This is likely to be at least partly due to the visual constraints of the eye, as longer words may require several fixations and thereby give decomposed stems a head-start. For short words, in turn, the whole complex word could potentially be read in one fixation, allowing direct activation of a full-form representation if such a representation exists. This finding is in line with compound word studies by Bertram and Hyönä (2003) and Andrews et al., (2004), as well as studies with inflected and derived words in French and English (e.g., Colé, Beauvillain, & Seguí, 1989; Beauvillain, 1996; Niswander, Pollatsek, & Rayner, 2000), reporting surface frequency effects for short complex words and lemma frequency effects for longer complex words.

The present results further indicate that readers predict upcoming morphs on the basis of the already seen ones. Prediction in recognition of morphologically complex words has been previously studied using the surprisal measure in phoneme prediction in the context of auditory words (Ettinger et al., 2014). There are also previous studies which suggest that variables that encode predictive information are influential in visual word recognition. For example, transitional probabilities from stem to suffix have been found to modulate lexical processing (Solomyak & Marantz, 2010; Lehtonen, Monahan, & Poeppel, 2011). To date, however, few cognitive models of morphological processing have explicitly taken within-word predictive information into account in their architecture.

## Conclusions

We utilized statistical models of morphology based on optimization to study the recognition of morphologically complex words. By utilizing eye-tracking, we looked into temporal characteristics of multimorphemic word processing, and by manipulating the task, we gained information about the task-specificity of particular effects. In sum, the MDL-based optimization principle which segments words at some morpheme boundaries while keeping others unsegmented was found to perform well in both tasks and both at early and later stages of word recognition, supporting dual-route cognitive models of morphological processing. However, morphological decomposition appears to dominate at early stages of word recognition. At later stages, the system utilizes information of morphological constituents and, additionally, predictive information of previous morphemes. This was, however, not the case for short words in which full-form processing may take place throughout the processing chain. At least in longer words, the performance of whole-word-based models seems to primarily stem from reanalysis, licensing, and decision-related processes that are emphasized in the standard lexical decision task. In more naturalistic conditions, these models’ prediction ability decreased. The results thus highlight the importance of considering task demands when studying morphological processing. They also demonstrate that the relative weight of the full-form and decomposition routes is strongly guided by word length.

## Appendix A: Correlations between word properties and language models

**Table 13:** Correlations between word properties and language models

	Number	Number	Lemma	Morphological	Word	Morfessor	Morfessor	Morfessor	Morfessor	Morph	Morph
	of letters	of morphs	frequency	family size	unigram	*α* = 0.01	*α* = 0.8	*α* = 10.0	CatMAP	unigram	bigram
Number of letters											
Number of morphs	0.63 ***										
Lemma frequency	− 0.05	0.13 *									
Morphological family size	− 0.02	0.24 ***	0.78 ***								
Word unigram	0.36 ***	0.39 ***	− 0.37 ***	− 0.31 ***							
Morfessor *α*= 0.01	0.62 ***	0.45 ***	− 0.38 ***	− 0.39 ***	0.54 ***						
Morfessor *α*= 0.8	0.56 ***	0.54 ***	− 0.38 ***	− 0.29 ***	0.74 ***	0.68 ***					
Morfessor *α*= 10.0	0.40 ***	0.44 ***	− 0.37 ***	− 0.33 ***	0.91 ***	0.57 ***	0.74 ***				
Morfessor CatMAP	0.47 ***	0.46 ***	− 0.38 ***	− 0.26 ***	0.71 ***	0.60 ***	0.88 ***	0.72 ***			
Morph unigram	0.66 ***	0.91 ***	− 0.11 *	− 0.01	0.54 ***	0.62 ***	0.69 ***	0.58 ***	0.62 ***		
Morph bigram	0.48 ***	0.59 ***	− 0.22 ***	− 0.21 ***	0.73 ***	0.61 ***	0.77 ***	0.73 ***	0.74 ***	0.73 ***	

*** *p* <.001. ** *p* <.01. * *p*<.05

## Appendix B: Descriptions of the statistical models of interest

### Word unigram

We used a word unigram model as a measure of the surface frequency of the stimulus words. The probability of a word form is then estimated based on its frequency in the training data. To prevent overfitting, we smoothed the maximum likelihood estimates with absolute discounting (Ney, Essen, & Kneser, 1994) and interpolation with uniform distribution:

1 $$ p(w) = \frac{max(c(w)-d,0)}{{\sum}_{w \in V} c(w)} + \frac{d \times | \{ w \in V : c(w) \geq d \} |}{{\sum}_{w \in V} c(w)} \times \frac{1}{|V|}, $$

where *c*(*w*) is the number of times w occurred in the training data, *d* ≥ 0 is the discount, and *V* is the vocabulary of the model. The smoothing parameters of the word unigram model were selected by optimizing the correlation between the predictions and the reaction times. The optimization was performed on an earlier reaction time data set reported in Lehtonen et al., (2007). A grid search provided the discount value *d* = 1.1.

### Morph n-grams

The segmentation of words to linguistic morphs was obtained by using the FINTWOL morphological analyzer (Lingsoft, Inc.). We utilized morph unigram and morph bigram models in the analyses. An n-gram model assumes that the next symbol is predicted based on the *n* − 1 previous symbols:

2 $$ p(w) \approx \sum\limits_{i=1}^N p(m_i | m_{i-n+1} ... m_{i-1}) $$

Here the symbols *m*_*i*_ for 0 < *i* < *N* are linguistic morphs. The initial and last symbols *m*_0_ and *m*_*N*_ are reserved for a word boundary symbol so that the model can predict when the word ends. For example, the English word “cats” would be estimated by a morph 2-gram model as

3 $$ p(cats) = p(cat | \#) \times p(s | cat) \times p(\# | s), $$

### Morfessor Baseline

Morfessor (Creutz and Lagus, 2002, 2005a, b, 2007) is a family of methods for unsupervised learning of morphological segmentation. Inspired by the MDL principle (Rissanen, 1978, 1989), modeling the language data in Morfessor is viewed as a problem of how to encode a data set efficiently in order to transmit it with a minimal number of bits. In two-part coding of a single parametric model, one first transmits the model parameters, and then the data set by referring to the parameters. Mathematically, the task is to find the parameters *𝜃* such that the two-part cost function L(*𝜃*, corpus) is minimized:

4 $$ \theta^{*} = \arg \min\limits_{\theta} L(\theta, \text{corpus}) = \arg \min\limits_{\theta} \{L(\theta) + L(\text{corpus}|\theta)\} $$

In the case of segmenting words into morphs, the model parameters simply consists of a lexicon of unique morphs, and a pointer assigned for each. The corpus is then transmitted by sending the pointer of each morph as they occur in the text.

The size of the training data has a direct effect on the size of the lexicon and the lengths of the units, and a larger corpus is not always better (Creutz & Lagus, 2007; Virpioja, Kohonen, & Lagus, 2011a). Instead of training the model on different corpora, it is simple to include a weight hyper-parameter *α* > 0 to the cost function (Kohonen, Virpioja, & Lagus, 2010; Virpioja et al., 2011a)

5 $$ \theta^{\alpha} = \arg \min\limits_{\theta} \{L(\theta) + \alpha \times L(corpus | \theta)\} $$

A large *α* will emphasize the likelihood part, leading to a large lexicon of long units. A small *α* will emphasize the prior and provide a small lexicon of short units. The two extremes correspond to a word unigram model and a letter unigram model. From the psycholinguistic viewpoint, low values of the likelihood weight are then associated with extensive morphological segmentation and high values with full-form processing. For training Morfessor Baseline models, the Morfessor 2.0 Python implementation by Virpioja, Smit, Grönroos, and Kurimo (2013) was used.

Note that while the word probabilities as estimated by the word unigram model and Morfessor with the corpus weight of 10.0 have a fairly high correlation coefficient (0.91), their values do not fully correspond. Even with this corpus weight setting, Morfessor still segments some words in the training data, leading to the differences between the models. Another difference may stem from the fact that models that allow segmentation include prediction for the end of the words (i.e., that the word ends) which is not needed in a model that starts from the assumption of only full-form units.

### Morfessor CatMAP

The Morfessor Categories Maximum a Posteriori (CatMAP) is a method for unsupervised morphological segmentation in the Morfessor family (Creutz & Lagus, 2005a, 2007). The method extends Morfessor Baseline by including a Prefix-Stem-Suffix Hidden Markov Model (HMM) - structure for the morph transitions. The segmentation is presented hierarchically and each morph may be tagged with the corresponding morph category.

The maximum a posteriori (MAP) -estimate to be maximized is:

6 $$ \begin{array}{@{}rcl@{}} &&\arg \max\limits_{lexicon} P(lexicon|corpus)\\ &&\quad= \arg \max\limits_{lexicon} P(corpus|lexicon) * P(lexicon), \end{array} $$

The probability of the lexicon is written as:

7 $$ P(lexicon) = M! \cdot \prod\limits_{i=1}^M \left[ P(meaning(\mu_i)) \cdot P(form(\mu_i)) \right], $$

where the probability of each morph *μ*_*i*_ has been divided into two separate parts, *meaning* and *form* (Creutz & Lagus, 2005a). The factor *M*! is explained by the fact that there are *M*! possible orderings of a set of *M* items and the lexicon is the same regardless of the order in which the *M* morphs emerged.

The probability of the segmented corpus is written as:

8 $$ \begin{array}{@{}rcl@{}} &&P(corpus|lexicon) = \\ &&\quad\prod\limits_{j=1}^W \left[ P(C_{j1}|C_{j0}) \prod\limits_{k=1}^{n_j} \left[ P(\mu_{jk} | C_{jk}) \cdot P(C_{j(k+1)}|C_{jk}\right] \right] \end{array} $$

The product is taken over the *W* words in the corpus (token count), which are each split into *n*_*j*_ morphs. The *k*^*t**h*^ morph in the *j*^*t**h*^ word, *μ*_*j**k*_, has been assigned a category *C*_*j**k*_, and the probability of the morph is the probability that the morph is emitted by the category, written as *P*(*μ*_*j**k*_|*C*_*j**k*_). Additionally there are transition probabilities *P*(*C*_*j*(*k*+ 1)_|*C*_*j**k*_) between the categories, where *C*_*j**k*_ denotes the category assigned to the *k*^*t**h*^ morph in the word, and *C*_*j*(*k*+ 1)_ denotes the category assigned to the following, or (*k* + 1)^*t**h*^, morph. The transition probabilities comprise transitions from a special word boundary category to the first morph in the word, *P*(*C*_*j*1_|*C*_*j*0_), as well as the transition from the last morph to a word boundary, $P(C_{j(n_j+1)} | C_{jn_j})$.

Due to the more accurate modeling of morph properties and the transitions, the Morfessor CatMAP in most cases achieves higher morphological segmentation accuracy compared to the Morfessor Baseline -approach (Creutz & Lagus, 2005a, 2007).

## Appendix D: Regression analyses with word unigram as a base predictor in Experiment 1

**Table 14:** AIC values, decrease in the regression model deviance and *p* values for the primary analysis of Experiment 2. AIC = Akaike Information Criterion; GD = Gaze duration; FFD = First fixation duration; GmF = GD minus FFD

Predictor	GD			FFD			GmF		
	AIC	Δ	*p* value	AIC	Δ	*p* value	AIC	Δ	*p* value
Word unigram	− 1092	−	−	7804	−	−	7426	−	−
+ Morfessor *α* = 0.01	− 1124	33.938	5.691e-09 (***)	7774	31.618	1.876e-08 (***)	7378	49.816	1.688e-12 (***)
+ Morfessor *α* = 0.8	− 1136	46.251	1.040e-11 (***)	7765	40.164	2.335e-10 (***)	7366	61.948	3.527e-15 (***)
+ Morfessor *α* = 10.0	− 1102	11.477	7.047e-04 (***)	7797	8.837	2.952e-03 (**)	7410	18.425	1.767e-05 (***)
+ Morfessor CatMAP	− 1126	35.941	2.034e-09 (***)	7788	17.465	2.927e-05 (***)	7390	37.630	8.550e-10 (***)
+ Morph unigram	− 1121	31.048	2.517e-08 (***)	7764	41.722	1.052e-10 (***)	7380	47.863	4.571e-12 (***)
+ Morph bigram	− 1127	36.636	1.424e-09 (***)	7783	22.993	1.626e-06 (***)	7378	49.670	1.819e-12 (***)

*** *p* <.001. ** *p* <.01. * *p*<.05

## Appendix E: Correlations between background variables and the eye-tracking measures for the long and short words in Experiment 1

**Table 15:** Correlations between background variables and the eye-tracking measures for the long words in Experiment 1. GD = Gaze duration; FFD = First fixation duration; GmF = GD minus FFD

Predictor	GD	FFD	GmF
Number of letters	0.462 (***)	− 0.348 (***)	0.526 (***)
Number of morphs	0.235 (***)	− 0.198 (**)	0.268 (***)
Lemma Frequency	−.316 (***)	0.027	− 0.303 (***)
Morphological family size	− 0.275 (***)	− 0.111	− 0.238 (***)
Word unigram	0.500 (***)	− 0.138 (*)	0.497 (***)
Morfessor *α*= 0.01	0.407 (***)	− 0.158 (*)	0.411 (***)
Morfessor *α*= 0.8	0.526 (***)	− 0.202 (**)	0.532 (***)
Morfessor *α*= 10.0	0.488 (***)	− 0.156 (*)	0.483 (***)
Morfessor CatMAP	0.528 (***)	− 0.178 (**)	0.517 (***)
Morph unigram	0.359 (***)	− 0.212 (***)	0.387 (***)
Morph bigram	0.503 (***)	− 0.163 (**)	0.502 (***)

*** *p* <.001. ** *p* <.01. * *p*<.05

**Table 16:** Correlations between background variables and the eye-tracking measures for the short words in Experiment 1. GD = Gaze duration; FFD = First fixation duration; GmF = GD minus FFD

Predictor	GD	FFD	GmF
Number of letters	0.184	− 0.517 (***)	0.240 (*)
Number of morphs	0.186	− 0.119	0.172
Lemma Frequency	− 0.394 (***)	− 0.084	− 0.408 (***)
Morphological family size	− 0.360 (***)	− 0.003	− 0.374 (***)
Word unigram	0.600 (***)	− 0.049	0.580 (***)
Morfessor *α*= 0.01	0.360 (***)	− 0.166	0.403 (***)
Morfessor *α*= 0.8	0.560 (***)	− 0.225 (*)	0.598 (***)
Morfessor *α*= 10.0	0.610 (***)	− 0.092	0.631 (***)
Morfessor CatMAP	0.511 (***)	− 0.146	0.530 (***)
Morph unigram	0.410 (***)	− 0.133	0.403 (***)
Morph bigram	0.576 (***)	− 0.206 (*)	0.635 (***)

*** *p* <.001. ** *p* <.01. * *p*<.05

## Appendix F: Regression analyses with word unigram as a base predictor in Experiment 2

**Table 17:** AIC values, decrease in the regression model deviance. and *p* values for the primary analysis of Experiment 2. AIC = Akaike Information Criterion; GD = Gaze duration; FFD = First fixation duration; GmF = First run GD minus FFD

Predictor	First run GD			FFD			GmF		
	AIC	Δ	*p* value	AIC	Δ	*p* value	AIC	Δ	*p* value
Word unigram	8176	−	−	3603	−	−	6855	−	−
+ Morfessor *α* = 0.01	8113	77.376	1.413e-18 (***)	3565	40.518	1.948e-10 (***)	6830	38.100	6.721e-10 (***)
+ Morfessor *α* = 0.8	8101	88.977	3.995e-21 (***)	3589	16.909	3.921e-05 (***)	6799	68.445	1.305e-16 (***)
+ Morfessor *α* = 10.0	8177	13.655	2.197e-04 (***)	3606	0.016	9.006e-01	6864	4.220	3.995e-02 (*)
+ Morfessor CatMAP	8142	48.650	3.059e-12 (***)	3600	6.166	1.302e-02 (*)	6824	43.976	3.325e-11 (***)
+ Morph unigram	8091	99.084	2.420e-23 (***)	3556	50.314	1.310e-12 (***)	6799	69.236	8.736e-17 (***)
+ Morph bigram	8133	57.770	2.947e-14 (***)	3591	15.075	1.033e-04 (***)	6824	43.977	3.323e-11 (***)

*** *p* <.001. ** *p* <.01. * *p*<.05

## Appendix G: Correlations between background variables and the eye-tracking measures for the long and short words in Experiment 2

**Table 18:** Correlations between background variables and the eye-tracking measures for the long words in Experiment 2. GD = First run gaze duration; FFD = First fixation duration; GmF = GD minus FFD

Predictor	GD	FFD	GmF
Number of letters	0.668 (***)	− 0.401 (***)	0.572 (***)
Number of morphs	0.348 (***)	− 0.198 (**)	0.352 (***)
Lemma Frequency	− 0.206 (***)	− 0.188 (**)	− 0.112
Morphological family size	− 0.180 (**)	− 0.184 (**)	− 0.104
Word unigram	0.418 (***)	− 0.015	0.396 (***)
Morfessor *α*= 0.01	0.423 (***)	− 0.149 (*)	0.325 (***)
Morfessor *α*= 0.8	0.523 (***)	− 0.030	0.493 (***)
Morfessor *α*= 10.0	0.431 (***)	0.007	0.409 (***)
Morfessor CatMAP	0.465 (***)	0.012	0.455 (***)
Morph unigram	0.434 (***)	− 0.193 (**)	0.417 (***)
Morph bigram	0.481 (***)	− 0.029	0.461 (***)

*** *p* <.001. ** *p* <.01. * *p*<.05

**Table 19:** Correlations between background variables and the eye-tracking measures for the short words in Experiment 2. GD = First run gaze duration; FFD = First fixation duration; GmF = GD minus FFD

Predictor	GD	FFD	GmF
Number of letters	0.655 (***)	0.272 (**)	0.127
Number of morphs	0.343 (***)	0.269 (**)	0.053
Lemma Frequency	− 0.133	− 0.188	0.004
Morphological family size	− 0.154	− 0.118	− 0.007
Word unigram	0.558 (***)	0.392 (***)	0.158
Morfessor *α*= 0.01	0.383 (***)	0.225 (*)	− 0.043
Morfessor *α*= 0.8	0.521 (***)	0.363 (***)	0.118
Morfessor *α*= 10.0	0.566 (***)	0.407 (***)	0.128
Morfessor CatMAP	0.520 (***)	0.359 (***)	0.124
Morph unigram	0.426 (***)	0.329 (***)	0.068
Morph bigram	0.521 (***)	0.304 (**)	0.102

*** *p* <.001. ** *p* <.01. * *p* <.05
